# Early detection of COPD in primary care -The Copenhagen COPD Screening Project

**DOI:** 10.1186/1471-2458-10-524

**Published:** 2010-09-01

**Authors:** Anne Marie Lyngsø, Vibeke Backer, Vibeke Gottlieb, Birgitte Nybo, Marianne S Østergaard, Anne Frølich

**Affiliations:** 1 Department of Integrated Healthcare, Bisbebjerg University Hospital, Copenhagen, Denmark; 2 Department of Pulmonary medicine, Bisbebjerg University Hospital, Copenhagen, Denmark; 3 Department of Health Services Research, Institute of General Medicine, Copenhagen University, Denmark

## Abstract

**Background:**

Chronic Obstructive Pulmonary Disease (COPD) is among the leading causes of death in the world, and further increases in the prevalence and mortality are predicted. Delay in diagnosing COPD appears frequently even though current consensus guidelines emphasize the importance of early detection of the disease. The aim of the present study is to evaluate the effectiveness of a screening programme in general practice.

**Methods/Design:**

Subjects aged 65 years and older registered with a General Practitioner (GP) in the eastern Copenhagen will receive a written invitation and a simple questionnaire focusing on risk factors and symptoms of COPD. Subjects who meet the following criteria will be encouraged to undergo spirometric testing at their GP: current smokers, former smokers, and subjects with no smoking history but who have dyspnea and/or chronic cough with sputum.

**Discussion:**

The Copenhagen COPD Screening Project evaluates the effectiveness of a two-stage screening program for COPD in general practice and provides important information on how to organize early detection of COPD in general practice in the future.

## Background

Chronic Obstructive Pulmonary Disease (COPD) is among the leading causes of chronic morbidity and mortality throughout the world. In 2002, COPD was the fifth leading cause of death in the world, and further increases in its prevalence and mortality are predicted [1-4]. COPD is characterised by a slowly progressing airflow limitation caused by chronic inflammation in the bronchioles. Early-stage COPD is often symptomless, although coughing with sputum production is common. Smoking is by far the most important risk factor, accounting for 85-90% of all cases [3,4]. According to recent studies, at least 25% of smokers without initial disease will have clinically significant COPD after 25 years [5-8].

Early detection of COPD is crucial for promoting smoking cessation and instituting pharmacological and non-pharmacological therapy before patients reach symptomatic and costly stages of disease. Smoking cessation is followed by a marked reduction in the irreversible accelerated decline in FEV_1 _(forced expiratory volume in first second) associated with the development of the disease [3,4]. Even though current consensus guidelines, such as the Global Initiative for Chronic Obstructive Lung Disease (GOLD) and other national recommendations, emphasize the importance of early detection, delay in diagnosing COPD is still common [3,9-11]. A recent Danish study shows that approximately 430,000 Danes suffer from COPD, and at least one third are undiagnosed [12]. This is consistent with other recent estimates indicating that 25% to 50% of patients in the western societies with clinically important disease are undetected or misdiagnosed [3]. In general, a delayed diagnosis of COPD results from the patient's gradual adaptation to decreasing lung function (patients delay) and, in some cases, lack of awareness of or response to the patient's symptoms from physicians (doctors delay) [13-15]. For instance, in a Dutch study, 74% of all subjects with symptoms or signs of COPD or asthma never consulted their GP for respiratory complaints, regardless of the severity of symptoms or lung function impairment [13].

The GOLD international guidelines for COPD, as well as national guidelines, advise spirometry as the gold standard for accurate and repeatable measurement of lung function. Primary care physicians are in an ideal position to detect early-stage COPD and perform spirometry to confirm the diagnosis [3,9,11]. The present study emphasizes the important role of the GP in the early diagnosis of COPD. In Denmark, 99% of citizens are listed with a GP, i.e. is having their own GP. A substantial number of citizens are having this specific GP for a long period of their life and only shift to another GP if they leave the area, disagreement happens or if the GP retires. The GPs play a key role within the Danish health system as the first point of contact for patients and the gatekeeper to hospitals, specialists, and other types of multidisciplinary care. This system supports the development of long-term relationships between patients and GPs and gives the latter the ability to perform preventive efforts, such as spirometry, free of charge [16,17].

### Current strategies to improve early detection

In previous studies, mainly two methods have been used to increase the rate of early diagnosis of COPD: screening of high-risk populations and case-finding. High-risk screening studies offer spirometry to all people in a selected population who meet certain criteria; often smokers, whereas case finding studies offer tests only to those who consult a GP for other health problems. Their advantages and disadvantages make them complementary approaches [18-24].

Effective case-finding depends on patient contact with GPs. In 2005, approximately 15% of the Danish population had no contact with their own or other GPs [25]. Although case finding of COPD in general practice has proven to be a more realistic approach than screening entire populations, it still leaves out a significant proportion of subjects at risk. Furthermore, case-finding assumes that every GP is fully aware of patients at risk for COPD and that they perform spirometry when needed. According to a recent study performed at Oesterbro, in eastern Copenhagen, 3,000 subjects above 65 years were at risk of COPD in 2007 [12]. However, only 224 (7.5%) spirometries were performed in 2007 among the 3000 citizens at risk. Furthermore these 224 spirometies were performed by one of the 56 General Practitioners at Oesterbro (Centralized GP observation), indicating that in average each GP perform four spirometries per year.

Screening and case-finding programs have mainly focused on smokers and former smokers. However, the present model also includes subjects with no smoking history, but with a history of dyspnoea and/or chronic cough with sputum [15,19,20,22-24,26-28].

### Aim of the study

The aim of the study is two-fold. First, we will assess if a short, mailed questionnaire, based on patient-reported information can serve as a first-level screening tool for the identification of individuals at risk for COPD. Secondly, we would like to assess if the self-administrated questionnaire can be used to prompt individuals at risk to undergo spirometric testing at their GP for an early diagnosis.

## Methods/design

### Setting and population

**Figure 1:**
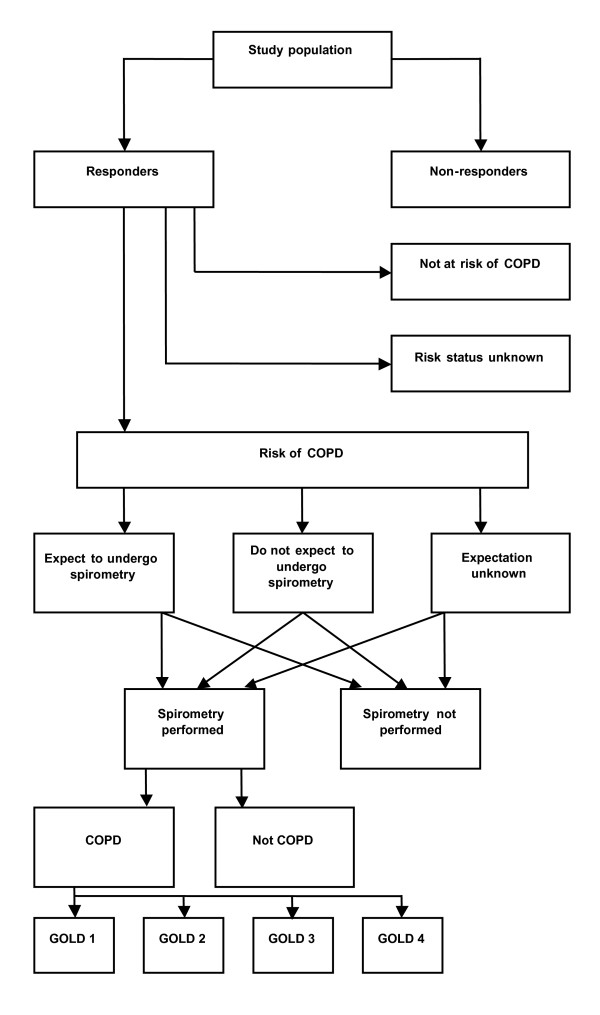
**Study flow chart**.

**Figure 2:**
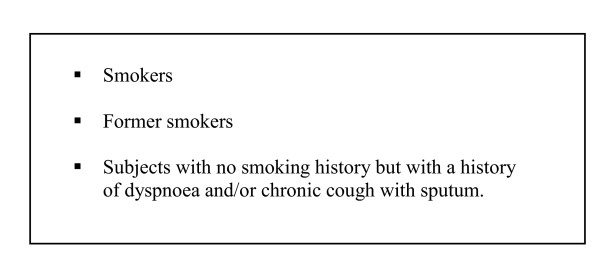
**Inclusion criteria for spirometry**.

This study is collaboration between the City administration of Copenhagen, Bispebjerg University Hospital, and GPs in the local area of Oesterbro. It will be performed in eastern Copenhagen, which has a population of 67,330 citizens and 56 GPs. All individuals, aged 65 years and older, registered with one of the 56 general practices will be selected for the study. The Capital Region will provide the subjects and their addresses from the Danish Civil Registration System (CPR), in which all Danish citizens have a personal identification number. All subjects from the study population will receive a short questionnaire in order to identify those at risk of COPD. The participating GPs will be asked to screen every subject at risk who requests an appointment for spirometric testing (Figure 1). Inclusion criteria are shown in Figure 2. Subjects will be excluded from the study if they are nursing home residents, since we expect that most of them will not be able to visit their GP without help. Subjects not registered with a general practice, or subjects who change from one GP to another after selection but before the invitation letters are mailed, will also be excluded. The number of excluded subjects within each of the above listed reasons will be counted.

### Interventions

#### Invitation to spirometry/questionnaire

**Figure 3:**
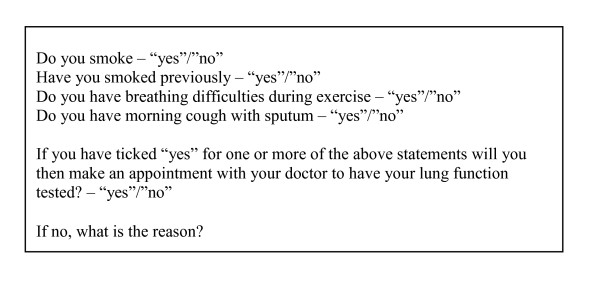
**Questionnaire**.

All subjects will receive an invitation letter by mail containing a small booklet about the study and general information about COPD, its prevalence, and the relation between the disease and smoking. They will be asked to complete a questionnaire indicating their smoking status and potential COPD symptoms (Figure 3). Subjects with at least one positive answer will be encouraged to undergo spirometry at their GP and asked to indicate whether they expect to contact their GP for an appointment. Subjects with no smoking history or symptoms will be asked to return the questionnaire with no further action. A preaddressed postage-paid envelope will be included. A first (and second) reminder will be sent to non-responders. Patients with a previous diagnosis of COPD will be invited to participate. To prevent excessive GP workloads, invitations will be sent in three rounds, and GPs will receive a list of patients receiving invitation letters.

To increase participation rates, advertisements will be placed in the local newspapers three times during the study period. The project manager will also visit local senior clubs to provide information on the project and to encourage subjects to participate.

#### Pulmonary function testing and diagnostic criteria of COPD

Spirometry will be performed using the different spirometers available in each practice; as not all GPs have a spirometer, an EasyOne^® ^spirometer will be available to each practice that needs one for the duration of the project. Spirometry will be performed by the GP or practice nurse/assistant. Subjects enrolled in a General Practice office that does not offer spirometry, are invited to contact the Health Care Centre in eastern Copenhagen for a lung function test. A specially trained project nurse will perform spirometry and forward results to the patient's GP, who is responsible for the interpretation of the test. All spirometry performed by GPs will be re-evaluated by an experienced physician at Department of Respiratory Medicine, Bispebjerg University Hospital. Written informed consent will be obtained from all subjects prior to spirometry.

Forced vital capacity (FVC) and forced expiratory volume in first second (FEV_1_) will be measured, and the FEV_1_/FVC-ratio will be calculated. The FVC and FEV1 used will be the largest value obtained from any of three technically satisfactory tests that vary no more than 5% or 100 ml, whichever is greater. Post-bronchodilator FEV1/FVC and FEV1 measurements will not be performed at this occasion.

Spirometry measurements will be evaluated by comparison to reference values based on age, height, sex, and race [29]. Spirometry results will be classified according to the GOLD guidelines (Table 1) [3]. It is the GPs responsibility to initiate medical treatment in patients diagnosed with COPD and, if necessary, to refer patients to a pulmonary rehabilitation programme in accordance with the local COPD disease management programme.

**Table 1:** Spirometric Classification of COPD Severity.

**Based on Post-Bronchodilator FEV**_**1**_	
Stage I: Mild	FEV_1_/FVC < 0,70 FEV_1 _≥ 80% predicted

Stage II: Moderate	FEV_1_/FVC < 0,70 50% ≤ FEV_1 _< 80% predicted

Stage III: Severe	FEV_1_/FVC < 0,70 30% ≤ FEV_1 _< 50% predicted

Stage IV: Very Severe	FEV_1_/FVC < 0,70 FEV_1 _< 30% predicted or FEV_1 _< 50% predicted plus chronic respiratory failure

According to a scale of fees agreed upon by the Organisation of General Practitioners and the Danish Regions, a special fee-for-service payment to the GP for performing spirometry will be posted. To create an economic incentive for participation, the payment will be slightly higher for performing spirometry for the study, as compared to usual compensation for spirometry. The higher payment will compensate GPs for required data collection. The patients will not receive any economic compensation.

#### Bispebjerg University Hospital

If the GP or the nurse at the health care centre is not able to carry out a proper test or finds it difficult to interpret the result, the patient will be referred to the outpatient clinic at the Department of Pulmonary Medicine at Bispebjerg University Hospital. At the clinic a specialized COPD nurse will perform the test and inform the patient and the patient's GP about the result.

#### Education seminar/ongoing support in general practice

Five studies have shown that implementation of spirometry in general practice is feasible if adequate training is used and interpretation of spirometry is provided [30-34]. However, a Danish study of 2549 patients shows that spirometry data documenting presence of airway obstruction are only available in 50% of patients diagnosed with COPD and that severity assessment (based on FEV_1 _% predicted value) is only conducted in 30% of patients diagnosed with COPD [35]. These results underscore the need for further education and support in the use of spirometry in general practice in Denmark.

Therefore, prior to the screening, all GPs and their clinical staff will be invited to participate in a two-hour course that includes aspects of diagnosis and management of COPD, as well as training in performance and interpretation of spirometry. Continuous technical and methodological support is available to the participating GPs through the screening period. The course and ongoing support will be carried out by a physician from the Department of Pulmonary Medicine, Bispebjerg University Hospital, and the project leader.

In addition to the course, all GPs will receive written instructions on how to perform spirometry and interpret results and on medical treatment and referral procedures for pulmonary rehabilitation in accordance to the GOLD-guidelines. They will also receive a list of names and identification numbers for patients invited to participate.

#### Home care Oesterbro

All nurses and home helpers at Home care Oesterbro will be invited to participate in a two-hour course that will include a short introduction to the project, information on symptoms of COPD, treatment, and how to help COPD patients to cope with daily life. The course will be carried out by a specialised nurse in COPD and the project leader. The purpose is to increase participation; patients receiving home care will typically be the weakest of the invited population and some may need help to respond to the invitation letter.

### Expected patient volume

**Figure 4:**
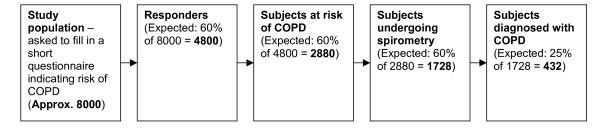
**Expected patient volume**.

A calculation of the expected patient volume is shown in figure 4.

### Outcome measures

To estimate the effectiveness of the questionnaire as a first-level screening tool for the identification of subjects at risk, we will look at the response rates to the questionnaire and the number of subjects at risk. Secondly, we will measure the interest in undergoing spirometry among high-risk subjects as measured by the answers to the questionnaire and the number of spirometries performed. In regard to the number of performed spirometries, we will compare participation rates among those who have been told to contact their GP and those who have been told to contact the local health care centre. Finally, we will assess the prevalence and severity of COPD among subjects who undergo spirometry.

### Planned analysis

To analyse the response rate to the questionnaire and the number of people at risk, descriptive statistics will be used. The association between individuals expectation to undergo spirometry and patient characteristics will be evaluated as well as the association between having spirometry performed and patient characteristics. Logistic regression will be used to estimate the odds of expected participation and actual participation (spirometry) with age, sex and smoking status as the independent variables. Comparisons between the group told to contact their GP for a test and the group told to contact the health care centre will be made with Chi-square tests. Finally the prevalence and severity of COPD will be estimated with descriptive statistics.

### Ethical approval

The study will be conducted according to the principles of the Helsinki declaration. The study is approved by the Danish Scientific Ethics Committee under ref. no H-B-2007-057, by the Danish Data Protection Agency and by the Committee of Multipractice Studies in General Practice under ref. no: MPU 16-2007.

### Project timeline

The study will be completed between February 2008 and February 2009.

## Discussion

The current study will be able to assess whether a short, mailed questionnaire based on patient-reported information can serve as a first-level screening tool for the identification of subjects at risk of COPD. Secondly it will be able to illustrate whether a focused questionnaire can prompt individuals at risk to undergo spirometric testing at their GP for an early diagnosis. It will be more economically efficient than broad population-level screening for COPD and reach a larger population than is possible through case-finding alone. By including not only smokers, but also former smokers and individuals with no history of smoking, but with a history of either dyspnea, chronic cough, or both, we will reach the largest possible population at risk.

## Competing interests

The authors declare that they have no competing interests.

## Authors' contributions

AML drafted the manuscript. AML, VB, VG, BN, MSØ and AF participated in the design of the study. AF and AML obtained funding for the project. AML, VB, VG, BN, MSØ and AF read, commented, and approved the final version of the manuscript.

## Pre-publication history

The pre-publication history for this paper can be accessed here:

http://www.biomedcentral.com/1471-2458/10/524/prepub
